# Establishing and Sustaining a Culture of Evidence-Based Practice: An Evaluation of Barriers and Facilitators to Implementing the Best Practice Spotlight Organization Program in the Australian Healthcare Context

**DOI:** 10.3390/healthcare7040142

**Published:** 2019-11-12

**Authors:** Greg Sharplin, Pam Adelson, Kate Kennedy, Nicola Williams, Roslyn Hewlett, Jackie Wood, Rob Bonner, Elizabeth Dabars, Marion Eckert

**Affiliations:** 1 Rosemary Bryant AO Research Centre, Division of Health Sciences, University of South Australia, Adelaide 5000, Australia; pam.adelson@unisa.edu.au (P.A.); Kate.Kennedy@unisa.edu.au (K.K.); marion.eckert@unisa.edu.au (M.E.); 2 Australian Nursing and Midwifery Federation (SA Branch), Ridleyton SA 5008, Australia; nicola.williams@anmfsa.org.au (N.W.); roslyn.hewlett@anmfsa.org.au (R.H.); jackie.wood@anmfsa.org.au (J.W.); rob.bonner@anmfsa.org.au (R.B.); elizabeth.dabars@anmfsa.org.au (E.D.)

**Keywords:** health service evaluation, implementation science, evidence-based practice, best practice guidelines, program evaluation, nursing and midwifery

## Abstract

Background: Nurses and midwives are central to the implementation and delivery of quality care through evidence-based practice (EBP). However, implementation of EBP in nursing and midwifery is under-researched with few examples of systematic and sustained change. The Registered Nurses Association of Ontario’s Best-Practice Spotlight Organization (BPSO) Program was adopted in South Australia as a framework to systematically implement EBP in two diverse and complex healthcare settings. Methods: The study was a post-implementation, mixed-method evaluation conducted at two healthcare settings in Adelaide, South Australia utilizing qualitative and quantitative data. Proctor’s implementation evaluation framework guided the evaluation design. Information sources included; interviews, focus groups, questionnaires, and document review. Results: Clinical and executive staff (*n* = 109 participants) from a broad range of stakeholder groups participated in the interviews, focus groups, and returned questionnaires. A number of facilitators directly affecting program implementation were identified; these pertained to embedding continuity into the program’s implementation and delivery, a robust governance structure, and executive sponsorship. Barriers to implementation were also identified. These barriers pertained to organizational or workforce challenges; staff turnover and movement (e.g., secondment), insufficient staff to allow people to attend training, and a lack of organizational commitment to the program, especially at an executive level. As a result of successful implementation, it was observed that over three years, the BPSO program positively influenced the uptake and implementation of EBP by clinicians and the organizations into which they were introduced. Conclusions: The BPSO model can be translocated to new healthcare systems and has the potential to act as a mechanism for establishing and sustaining EBP change. This study was the first to apply an implementation evaluation framework to the BPSO program, which allowed for structured analysis of facilitating or impeding factors that affected implementation success. The findings have important implications for other health systems looking to translocate the same or similar EBP programs, as well as contributing to the growing body of implementation evaluation literature.

## 1. Introduction

Nurses and midwives are at the forefront of providing high quality, patient-centered care and ensuring patient safety. In Australia, they are the largest group of healthcare practitioners representing 57% of the total healthcare workforce [1,2]. The majority (93%) of these nurses and midwives work in clinical care; in hospitals, residential health care facilities, and community health care services [3], directly affecting the quality and safety of healthcare delivered in Australia.

Australia’s hospitals are a mixed system of approximately 65% public beds and 35 % private. It is a complicated system with different levels of government responsible for funding, service provision, education, and training. Operating within this system are different levels of organizational culture, leadership, and existing models of care [4]. This complexity introduces challenges with respect to adopting and sustaining a nursing and midwifery workforce culture focused on quality improvement.

The Australian Commission on Safety and Quality in Health Care is the Australian organization responsible for the establishment of and updates to National Safety and Quality in Health Service (NSQHS) Standards. The Commission aims to support quality improvement initiatives by providing a safety and quality framework for all hospitals and healthcare facilities. This framework is mandated by Health Ministers and linked to hospital accreditation. The NSQHS were updated in 2017 to eight Standards [5], many of which are nursing sensitive. Successful implementation of these standards relies on a health care organization’s ability to demonstrate evidence-based practice (EBP), client-centered care, and consumer engagement.

Evidence-based practice (EBP) is the interrelationship between the best available evidence, clinical expertise, the health service context, and the patient’s needs, values, and preferences for their treatment and care [6,7,8]. EBP leads to high-quality care, improved patient outcomes, and reduced costs. However, sustaining EBP is not straightforward and many barriers prevent clinicians from consistently implementing EBP including inadequate skills and knowledge [8,9,10]

Nurses and midwives play a critical role in bringing about EBP change, and although they understand the value of EBP, many perceive their own knowledge and skill in the area as insufficient [9]. Indeed, the successful implementation of EBP is dependent on a number of variables including individual experiences, professional, organizational, and workforce factors [10,11]. Furthermore, the translation, uptake, measurement, and evaluation of EBP into the clinical environment and presents many challenges [12], which can unravel efforts to sustain and demonstrate EBP change.

### 1.1. The Best Practice Spotlight Organization (BPSO®) Program

Implementing and sustaining EBP in healthcare settings whether at the hospital, network or system-wide level requires a foundation on which to do this. Professional nursing organizations throughout the world, but in particular in North America, the UK, Europe, and Australia have served as peer leaders in the promotion of EBP among nurses, and the development of EBP programs and best practice guidelines (BPGs) [13]. BPGs are documents that support EBP through synthesis of the best available research evidence with specific recommendations for clinical decision-making [14].

The Best Practice Spotlight Organization (BPSO) Program was first launched in Ontario in 2003 and was developed by the Registered Nurses Association of Ontario (RNAO) as part of its nursing BPG program [15,16]. The program currently operates in over 15 countries and has been successful in demonstrating the uptake and utilization of best practice guidelines. It is listed as a resource centre for nursing guidelines on the Guidelines International Network (G-I-N). BPSOs are healthcare organizations and academic institutions that have formed a partnership with the RNAO to implement and evaluate selected RNAOs best practice guidelines. The process of becoming a BPSO designate organization involves a competitive application process and once organizations successfully complete the three-year pre-designate period (previously referred to as candidacy period), they can achieve BPSO designation [11]. Each guideline provides best practice recommendations in three main areas; at the practice level for nurses and other health-care providers, at the system and organizational level, and at the educational level for those responsible for staff and student education.

Implementation of EBP through the RNAO framework is based on the knowledge-to-action framework; a process model that follows specific stages in the process of translating research into practice, including the implementation, diffusion, and dissemination of research into various implementation aspects [17]. Critical to implementing nursing BPGs as part of the BPSO program is the role of nurse champions. This involves nurses and other health-care professionals participating in orientation workshops designed to provide them with strategies to champion BPGs in their organization and form the best practice champions Network. The champions model is established in the RNAO literature as an effective model of knowledge transfer to incorporate evidence into practice [18,19].

### 1.2. The BPSO Program in Australia

In South Australia, where this study is based, EBP initiatives have been attempted over many years. The Australian Nursing and Midwifery Federation (ANMF; SA Branch) is an organization committed to supporting nurse- and midwife-led EBP. Through the organization’s efforts to improve sustained EBP change in SA, the ANMF (SA Branch) engaged with the Registered Nurses’ Association of Ontario regarding the BPSO Program. In 2012, SA became the first host internationally of the BPSO Program through a contractual agreement between the RNAO, the ANMF (SA Branch) and the South Australian Government Department of Health. The program was adopted to the SA public healthcare sector with the goals of:

- Establishing dynamic, long-term partnerships (international and local) that focus on patient care via knowledge-based nursing/midwifery practice;
- Identification of effective strategies for system-wide disseminating of best practice guidelines (BPG) implementation;
- Demonstrate creative strategies for successfully implementing nursing and midwifery best practice guidelines (BPGs) at both individual and organizational levels;
- Inaugurate effective evaluation protocols using structural, process and outcome indicators;
- Ensure sustainability and long-term integration of the program by creating attitudinal change among staff and therefore positively changing the culture of the organization.

The selection of the BPSO program framework to the Australian context was influenced by several factors including; the adaptability of the program to the local context, alignment with state and national initiatives (e.g., national safety and quality reporting), recognizing the role of positive practice environments, and the support of professional organizations and leaders in bringing about change. 

### 1.3. Evaluation of the Study Aim

Despite growing recognition of the value of EBP to support the advancement of nursing- and midwifery-led care, there has been little research evaluating the barriers and facilitators to implementing programs designed to support the adoption and sustainment of EBP into healthcare settings [20,21,22]. Few studies have assessed a holistic systems approach to engaging nurses and midwives as lead change agents in embedding EBP [11]. This study is unique in that it examines the structures, processes, and outcomes that enabled Australia to translocate the entire BPSO program model to a new healthcare context. Specifically, this study aimed to evaluate and describe key factors that affected implementing the BPSO program and the adopted BPGs in a complex healthcare setting, including the program’s long-term sustainability. The objectives of the study were to: (i) identify program-level factors that acted as facilitators or barriers to implementation success, (ii) describe how program implementation influenced downstream service delivery, and (iii) map this information against a prescribed set of implementation outcome and service outcome domains.

The study was conducted in accordance with the National Health and Medical Research Council guidelines for the conduct of evaluation studies in healthcare settings. Ethical approval for the BPSO program was obtained through the SA Health Human Research Ethics Committee (HREC-14-SAH-21). 

## 2. Materials and Methods

### 2.1. Context of the Research

South Australia (SA) is one of eight Australian states and territories with approximately 80% of the population living in the catchment area of its capital city, Adelaide. At the time of the evaluation, there were five local health networks (LHNs); four based in the Adelaide metropolitan region, and one across regional South Australia. These LHNs manage the delivery of public hospital and community services in SA. The study was conducted at two sites within two of these LHNs. Each site was required to submit a proposal to participate in the BPSO program. In 2015, two sites were selected through a competitive application process to participate in the BPSO program as pre-designate sites from 2016–2018: Site 1 was focused specifically on the care of women and pediatrics and Site 2 was a directorate focused on caring for people living with mental health issues (Table 1). 

### 2.2. Conceptual Framework

**Figure 1:**
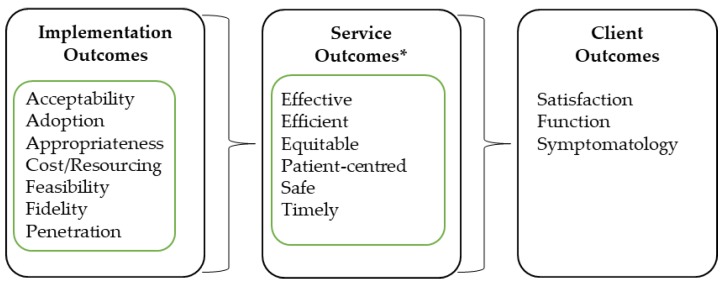
Proctor’s conceptual framework for evaluating implementation of innovations in healthcare settings [23]. *Service outcomes align to the Institute of Medicine (IOM) Standards of Care [25].

The study was defined and undertaken as a post-implementation, mixed-method evaluation from the perspective of the health care provider. The evaluation design was structured to align to the evaluation framework for implementation outcomes developed by Proctor and colleagues [23,24]. Proctor’s framework was identified as the most suitable conceptual framework to guide the overall study design because it was specifically developed for evaluation of implementation activities within the context of health care. The framework structures data collection related to the effectiveness of the implementation process with respect to specific outcome domains and relates these to downstream service and client outcome domains (Figure 1). This study focused on the implementation- and service-level outcomes only.

### 2.3. Participants

Participants were recruited using three methods: interviews, focus groups, or via an online questionnaire. Participants selected for interview or focus group attendance were determined based on stakeholder positions from the BPSO program governance structure including senior nursing staff at each LHN (e.g., the office of the Director of Nursing/Midwifery, Co-Director of Nursing/Midwifery, and Nursing Directors), BPSO steering committee representatives, BPSO site leads, BPSO site champions, safety and quality coordinators, other LHN nursing and midwifery and multidisciplinary staff, and consumer and community engagement representative/s, the ANMF (SA Branch), and office of the Chief Nurse/Midwife. 

Staff within each site were invited to participate in an online questionnaire, which allowed the researchers to evaluate the perceptions of staff from outside of the key stakeholder group with respect to the BPSO program at their site. This allowed for corroboration of interview findings or identified areas of discrepancy.

### 2.4. Data Collection Tools

The study included an analysis of routinely collected program data collected by each site over the three-year pre-designate period, review of BPSO key resources (e.g. BPGs, implementation guidelines, and LHN governance structure), and questionnaires and interviews with key stakeholders.

#### 2.4.1. Routinely Collected BPSO Program Evaluation and Reporting Data

The routinely collected BPSO data used to monitor and evaluate the success of the pre-designate sites against the BPSO program objectives was provided by the sites included in the study. These data incorporated qualitative and quantitative indicator data and measurements aligned to the LHN’s policy, safety and quality, and reform agendas.

#### 2.4.2. Review of Key Documentation

Additional documents provided by sites (e.g., progress reports) were reviewed according to the objectives. Key documents included BPSO program resources pertinent to each LHN along with those resources developed by each LHN, ANMF (SA Branch), and RNAO to support program implementation.

#### 2.4.3. Interviews and Focus Groups

Interviews and focus groups were used to collect data relevant to stakeholders’ experiences of the BPSO program and the dissemination and adoption of BPGs within the LHN. These were coordinated in collaboration with the ANMF (SA Branch) and the LHNs to ensure key stakeholders within LHNs are present. The interviews and focus groups followed a semi-structured format with prompt questions provided to participants captured under the four main questions of the evaluation. Introductory questions were used to capture basic demographics and experience with the BPSO program.

#### 2.4.4. Questionnaire

A questionnaire was developed to gather self-reported feedback from staff at the two sites regarding implementation of the BPSO program including staff perceptions of barriers and facilitators. The questionnaire included a mixture of dichotomous (yes/no), Likert-type responses (e.g., strongly agree through to strongly disagree), and short open-ended questions. The questionnaire was modified slightly for each site to suit the local context (e.g., introductory text) and administered electronically via SurveyMonkey to staff members within LHNs with varying levels of exposure and engagement with the BPSO program. The questionnaire was opened from 23 November to 12 December 2018.

The questionnaire included questions related to people’s level of awareness of the BPSO program overall and that their site was a pre-designate site. The questionnaire also included a 14-item assessment of how conducive the workplace environment was to support the implementation of an EBP program (like the BPSO program) using a four-point, Likert-type scale (e.g., strongly agree, agree, disagree, and strongly disagree). Examples of questions included “Senior staff actively encourage staff to participate in BPG training”, “We are encouraged to pursue continual quality improvement”, and “We have good teamwork between all clinical disciplines”.

### 2.5. Procedure

The study was conducted between 8 November 2018 and 14 December 2018. Interview participants were invited via email or telephone. Some interviews were conducted together at the request of the interviewee. Two researchers were present for each interview, except for one interview. 

Focus groups were coordinated with the support of each site. BPSO site leads were responsible for identifying and supporting communication to attendees. It was understood by the researchers that those invited were people familiar with and engaged with the BPSO program at each site (e.g., site champions). Notes and audio recordings were taken at each interview and focus group for subsequent analysis.

### 2.6. Data Analysis

Data synthesis and analysis relied on triangulation of the data collated from the multiple data collection tools described above. Qualitative data was the primary type of data relied upon for this evaluation study with quantitative data used to describe participants and to verify qualitative information reported (e.g., proportion of BPSO champions trained within a site). Qualitative data (e.g., interviews, focus groups, open-ended questionnaire responses, and narrative text included in BPSO site reports) were analyzed using a deductive thematic analysis approach [26] aligned to Proctor’s framework.

Quantitative data (e.g., routinely collected BPSO program data, and questionnaire responses) were analyzed using descriptive analysis techniques including counts and frequency statistics. Crosstabs with Chi-square statistics were used to compare the two site questionnaires’ workplace environment responses (strongly agree/agree vs. strongly disagree/disagree).

## 3. Results

### 3.1. Participant Demographics

#### 3.1.1. Interview Participants

In total, 16 people were invited for interviews regarding the BPSO pre-designate site experience and 11 people accepted (Table 2). While efforts were made to meet with the outstanding interviewees, due to unforeseen events that interrupted scheduled meeting times, the remaining individuals were unable to be interviewed during the data collection period.

#### 3.1.2. Focus Group Participants

Four focus groups were originally planned (two per site) in order to cover a representation of staff engaged in the BPSO program outside of the key stakeholder groups, to seek their perspectives on its implementation and effectiveness within each site. However, after discussion with the BPSO pre-designate site leads, due to the difficulty of finding a time when a sufficient number of staff could attend, only one focus group was conducted per site with as many people encouraged to attend as possible. In total, 12 people attended across the two sites (Table 2).

#### 3.1.3. Questionnaire Participants

In total, 53 people from Site 1 and 37 from Site 2 commenced the online questionnaires. Four people (two from each site) were removed from analyses because they did not complete beyond the first page of questions (demographics); resulting in 51 from Site 1 and 35 from Site 2, respectively. In addition, not all people completed all questions, so the number of respondents varied per question.

With respect to questionnaire respondents, a higher proportion of females completed the survey (98% from Site 1 and 69% from Site 2). While there was some deviation in age range distribution of respondents from each site, the majority of people (70%) from both sites were aged over 45 years. There was some observable difference in the length of service of respondents at each of the BPSO pre-designate sites, with people from Site 1 reporting a longer length of service (73% vs. 35% >10 years’ service). The majority of staff from Site 1 were registered nurses (RN; 59%), registered midwives (RM; 12%), or RN/RM (24%). Site 2 respondents were more varied with 58% RN, 3% RM, and 39% other from allied health, medical, and administration roles.

Site 1 respondents reported high levels of awareness of the BPSO program overall (94%) and awareness that Site 1 was a pre-designate site (92%). Site 2 respondents reported moderate levels of awareness of the BPSO program overall (54%) and that Site 2 was a pre-designate site (42%).

### 3.2. Barriers and Facilitators to Effective Implementation of the BPSO Program in a Complex Health Care System

With respect to implementation outcomes, it was observed that a number of facilitators directly affected program implementation; these mainly pertained to embedding continuity into the program’s implementation and delivery (e.g., through structured and stable governance arrangements, stable association with units and personnel, and linked resources). It was reported that having an executive responsible for the program implementation and delivery was a critical success factor. In addition, the champions network was noted by interviewees and questionnaire respondents as an important mechanism for engaging all levels of employees and tangibly linking them using the champion title.

Barriers to implementation were also identified. These barriers predominantly related to organizational or workforce challenges; staff turnover and movement (e.g., secondment), not enough staff to allow people to attend training, and lack of organizational commitment to the program, especially at an executive level. The origins (nursing) and language of the BPSO program, which affected other disciplines initial engagement with the program (Table 3).

**Figure 2:**
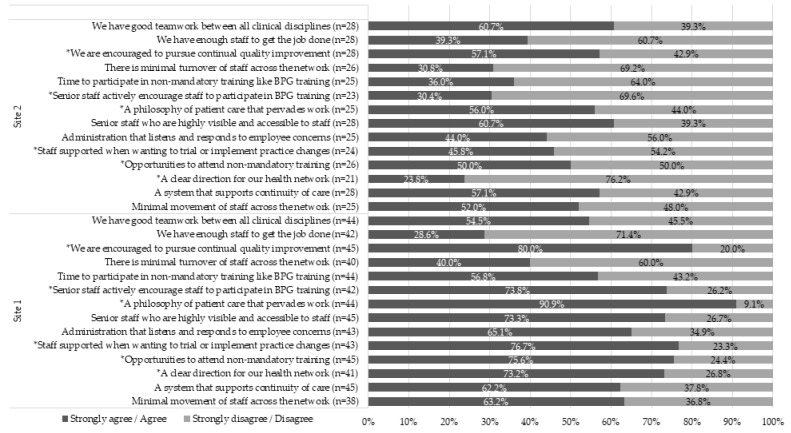
Staff perceptions of workplace environment factors conducive to implementing an EBP program, by site. * indicates significant difference between sites.

Staff perceptions of how conducive the workplace environment was to support the implementation of an EBP program showed that Site 1 had consistently more positive responses than Site 2 (Figure 2). Site 1 was more likely to have; “A clear direction for our health network” (χ^2^ = 13.76, *p* < 0.001), “Opportunities are provided to attend non-mandatory training” (χ^2^ = 4.81, *p* = 0.028), “Staff are supported when wanting to trial or implement practice changes” (χ^2^ = 6.53, *p* = 0.011), “A philosophy of patient care that pervades work” (χ^2^ = 11.42, *p* = 0.001), “Senior staff actively encourage staff to participate in BPG training” (χ^2^ = 11.51, *p* = 0.001), and “We are encouraged to pursue continual quality improvement” (χ^2^ = 4.40, *p* = 0.036). There were only two questions where Site 1’s responses showed a less positive trend, but these differences were not significant.

### 3.3. Impact on Service Delivery of the BPSO Program

Table 4 outlines examples of how service delivery was affected through successful implementation of the program, and where challenges were observed through difficult implementation. As a result of successful implementation, it was observed that the BPSO program had the potential to affect service delivery during its pre-designate period, even given the lag time consumed by planning and negotiation implementing the program and each BPG at each site. In some instances, it was reported that this time was important for ensuring successful execution of activities aligned to the BPG. For example, at Site 1, the ‘Woman’s abuse’ BPG was selected as an important, strategically aligned BPG for the site to adopt as part of its pre-designate period. The requirement for this BPG as a measure of success was that all women passing through the service would be screened for domestic violence using a routinely administered set of questions. This presented some challenges including having all clinical disciplines agree to and actively participating in this approach. Hence, there was a resultant lag-time, but the outcome noted during interviews was that this allowed an environment to mature for implementation of this BPG, which consequently affected the care received.

Where challenges to implementation were observed, the program still demonstrated some early indication of service changes with respect to initiatives led or supported under the auspices of the BPSO program. However, due to the implementation challenges, downstream impact on service outcomes was not as evident and not observable at a site-wide level. There were some specific examples described where there had been a concerted effort and investment towards implementation of an innovation, but these were isolated and it was not evident that the program was an instrumental factor in their adoption within the healthcare setting. For example, Site 2 was rolling out a program as part of its “Alternative use of restraints” BPG that replaced use of security guards with health worker assistants (HWAs) who were provided with an activity box. This approach was designed to engage the patient more humanely. While commendable for its objectives and potential to impact service delivery with respect to ‘patient-centered’ and ‘efficient’ (HWAs are cheaper than security guards), it was the implementation difficulties related to changing senior management and releasing staff for training to this BPG that affected its implementation penetration over three years.

## 4. Discussion

The aim of this study was to report on the implementation outcomes of the BPSO program at two BPSO pre-designate sites within a complex health service context and to illustrate how implementation affected service outcomes using examples from these sites. The first three years of a BPSO program at a site presented a unique opportunity to observe factors that affected the implementation of the program and allowed for identification of those factors critical to its success. This is important when considering translocation of programs to new settings [27]. This study was the first to apply an implementation evaluation framework to an EBP program, and complements other similar research focused on identifying factors salient to EBP implementation and sustainability [10,11]. 

### 4.1. Facilitators and Barriers to Implementation and Impact on Service Delivery

Proctor’s framework allowed for structured evaluation of the BPSO program implementation performance with respect to identifying facilitators and barriers to implementation. Both sites considered broader strategic initiatives being progressed within their site, which was a strategically wise decision when selecting the three BPGs as part of their pre-designate period. For example, the SA Health suicide prevention plan, published by the Office of the Chief Psychiatrist, was closely aligned to activities undertaken within Site 2 (e.g., connecting with people training). This facilitated many of the implementation outcome domains including acceptability, adoption, feasibility, and sustainability. However, this also made it difficult to directly ascribe all activities to initiatives being progressed under the banner of the BPSO program. This was noted for other BPGs, and potentially, exemplifies that alignment to broader initiatives is required, especially when attempting systemic change and/or a difficult BPG (e.g., Woman’s abuse).

Partnerships between units were also critical to success. For example, at Site 1 the partnership of the site BPSO program lead unit (the clinical practice development unit) with the consumer and community engagement unit was critical. The BPSO program’s service delivery was greatly augmented through this partnership with many people commenting on the value of having consumer presence and voice at training. In addition, many of their BPSO activities were directly aligned with existing works of the Consumer and Community Engagement Unit, which reduced duplication of effort. 

Extensive documentation of organizational level activities related to the BPSO program and changes helped to support the demonstration of the program’s impact, as well as efficiently building in program accountability and recognition. These included activity reporting, evaluation of Champion’s training, consumer and community engagement strategies, practice level activities, and changes and awards that have occurred against each BPG. 

The organizational commitment at the site, and the commitment of the external parties—in this instance ANMF (SA Branch), SA Health, and the RNAO—who each had roles to play to support the site, was critical to implementation success. Senior stakeholders interviewed commented that, given the numerous pressures a large healthcare providers faces in Australia and therefore the potential risk the organization is taking when committing to an organization-wide clinical quality improvement program like the BPSO program, understanding and communicating the benefits such a program would bring to the organization, and reinforcing this with evidence as the program rolled-out was necessary to maintain this commitment. Financing the BPSO program also supported this commitment. The financing arrangement in this instance was a three-way commitment between the three South Australian parties, and so there was internal and external interest in its success. In this sense, the funds acted more than just as money to pay for people and activities. It gave the program a budget line in the organization, it represented a financial risk to multiple parties, and combined gave the program greater substance. 

Champions training and the Best Practice Champions Network were vital elements in the uptake and embedding of EBP across organizations. While nurses may have positive attitudes and beliefs toward EBP, their beliefs are associated with the extent to which EBP is implemented [28]. The BPSO Program received many positive comments from nurses on its value, adaptability, and relevance to their organization and the South Australian healthcare context.

Implementation barriers were not always the antithesis of facilitators. While it was observed that factors critical to implementation success were also barriers when not in place (e.g., executive stability), there were also other barriers identified that presented separate challenges. As noted, there was resistance amongst clinicians to the introduction of the Woman’s Abuse BPG at Site 1. This was in part due to uncertainties regarding screening and responsibilities of clinicians around this sensitive area. Consequently, it took this BPG considerably longer to bring to a mature training and practice improvement program compared with the two other BPGs adopted by Site 1.

### 4.2. Implications for Clinical Practice

The results of this study provide program and service-level considerations related to BPG and EBP program implementation, as well as sustained practice change. First, this study reinforces previous research that there is no single solution to the application of EBP in healthcare [29], but that theoretically driven, structured programs, like the BPSO program, facilitate this process [30]. This study identified that strategic alignment of BPGs to service initiatives, embedding of the program that supports their implementation, and minimizing duplication of effort are key to the long-term sustainability and maintenance of EBP. The study showed that positive workplace environments influence uptake and application of BPG in the provision of safe, quality care, similar to findings from previous research [31]. Clinically focused BPGs are less likely to succeed in the long term without the organizational culture and processes to support them. For example, governance of BPGs being embedded in established clinical and corporate procedures, network-wide audits in collaboration with quality and safety representatives, consumer engagement activities, maintaining BPSO and BPG communication, and embedding the BPSO program into staff orientation and other courses.

Second, the study identified that the BPSO program was more than just a ‘vehicle’ to implement BPGs; its structure was acting as an ‘anchor’ for establishing and sustaining EBP change in the healthcare setting. In a stable, more positive workplace environment (e.g., Site 1), this assisted with supporting continual quality improvement and provided a vehicle for sites to improve organization-wide policy and evidence-based practice. In an organizational environment undergoing continual, widespread, and/or significant reform (e.g., Site 2), the program acted as a stabilizing function to maintain course direction towards continual quality improvement of safe and quality care. This is not to be underestimated when there may be changes in one or more levels of leadership, budget cuts, or changes in the political landscape that may result in decisions that impact on practice improvement programs.

Third, this study demonstrated that non-discipline specific change to improve the safety and quality of healthcare could be implemented irrespective of which discipline is nominally ‘leading’ it. As the single largest providers of health care in Australia, nurses have the potential to significantly influence and address gaps in EBP. While some have questioned the need for discipline-specific guidelines for EBP, it is the responsibility of members across all disciplines to be informed by best available research and some of this is discipline-specific [32]. However, challenges do exist when moving EBP across discipline boundaries, which as evidenced in this study, require other facilitating factors to be present in the environment in order to succeed (e.g., executive sponsorship, strategic alignment to policy, and time for discussion and negotiation). This is important when health services are planning large change; careful planning before acting, negotiation with and on-boarding of all stakeholders, especially those key influencers, can save significant time and organizational disruption during the ‘roll-out’ phase.

Fourth, the study highlighted the need for measurement and reporting as part of program success. Implementation of BPGs is a legal directive as well as a professional requirement in Canada [33] and measurement and reporting are embedded as part of the program through a standardized database [30]. As part of this study, interviewees responsible for monitoring and reporting the BPSO program noted that for each BPG well-defined and sensical (to the healthcare system) KPIs facilitated implementation and subsequent sustainability. However, when such KPIs were lacking, it presented a challenge for BPG implementation. In Australia, the development of the NSQHS standards has given rise to a number of developments to address the lack of reliable data for monitoring the quality of care and patient safety [34]. This presents an opportunity to closely align BPG KPIs to NSQHS standards, given that many of which are nurse sensitive. However, nurse sensitive indicators, while increasingly accepted that they assess nursing and midwifery’s impact on patients’ safety and health status [35], are not standardized and can vary in definition and measurement between organizations. The BPSO program provided a structured framework for promoting nurse or midwife-led EBP, their impact on patient safety and quality of care, and aligning BPG monitoring across organizations to nurse sensitive indicators, which served a dual purpose of improving care and satisfying NSQHS reporting requirements.

### 4.3. Implications for Implementation Evaluation

This study also served to demonstrate the practical application of an implementation framework to guide the structuring of the data analysis and reporting. Use of Proctor’s framework was important for aligning service outcomes to indices meaningful to healthcare services, especially as it relates to hospital accreditation safety and quality standards. This top-down approach focused the data analysis process on extracting only those bits of information relevant to the pre-defined domains. In an environment that is awash with data (i.e., the health system), this expedited the data extraction and reporting process, which may serve others interested in reviewing programs in their healthcare settings.

The IOM standards are quite broad terms, and are agnostic to the health system, so are useful outcomes to report against. However, it may be more suitable to use system context-specific measure of quality and safety to demonstrate benefit locally (e.g., using the Australian NSQHS standards to align with accreditation).

### 4.4. Study Limitations

The main limitation to the study was the limited amount of raw, quantitative data available to complement other data sources, as part of assessing the implementation outcomes and service outcomes. For example, it was not possible to extract firm evidence regarding impact on health service efficiency. While this is not a priority of the BPSO program (i.e., to have an explicit strategy to improve system efficiency), the cost of healthcare delivery is an important component to capture, especially if planning to affect large-scale change. Similar rationale could be argued for the other service outcome domains.

Greater emphasis was placed on qualitative data sourced from staff perceptions, perspectives, and experiences with respect to the implementation of the BPSO program, and how it affected the healthcare delivery. This was in part due to the nature of the methodological design and analysis, and the inherent limitations of having a small number of questionnaire respondents meaning less exploratory analysis were possible (e.g., meaningful assessment of work environment outcomes between those aware of vs. not aware of the BPSO program).

Response bias was a risk given the stake or interest many of the participants had, especially those who were interviewed or part of the focus group. The questionnaire assisted here by gathering feedback from people with very little awareness of or interest in the program. However, the questionnaire results should not be viewed as a generalizable representation of each site, and likely has other biases.

## 5. Conclusions

This research has added to the body of scholarship on enhancing and sustaining EBP programs in nursing and midwifery, and in health services more broadly. The research identified and described key implementation factors that facilitate successful implementation of a program, or are indeed, barriers to its implementation, designed to support the translation and embedding of EBP into healthcare. Furthermore, the research demonstrated that such programs can be translocated to a different health system context with moderate adaptations, but where careful planning and adherence to the program’s underlying theory is critical to sustained success. 

## Figures and Tables

**Table 1:** Characteristics of sites included in the study.

Headline	Site 1	Site 2
Patient population	Women and Children under 18 years	People living with mental health issues
BPSO pre-designate period	March 2016–March 2019	March 2016–March 2019
BPGs selected for the site (in order of implementation)	Person and Family Centered Care (PFCC) Care transitions Woman’s abuse	Alternative approaches to the use of restraint Assessment and care of adults at risk for suicidal ideation and behavior PFCC

**Table 2:** Number of invited and interviewed participants from four broad stakeholder groups.

Stakeholder Group	Invited for Interview	Interviewed	Focus Group Attendance
Site 1	6	4	8
Site 2	6	5	4
Host organization for the BPSO	3	2	NA
Office of Chief Nurse/Midwife	1	0	NA

**Table 3:** Description of site implementation of the Best Practice Spotlight Organization (BPSO) program with respect to Proctor’s implementation domains.

Implementation Outcome Domain	Facilitators with Respect to the Implementation Outcome Domain	Barriers with Respect to the Implementation Outcome Domain
Acceptability	Acceptability was positively impacted by: • BPGs being context independent, which made their adoption flexible and adaptable to the system context • Conducting training surveys to understand how participants perceived the training and modifying or course-correcting where required	Acceptability was negatively impacted by: • The language of the BPSO program, which added new terms and acronyms to learn in an environment that was already flooded. • Differing levels of “buy-in” from the executive leadership. • Disrupted leadership at multiple levels, which had the potential to disrupt the program implementation. • Complexity and sensitivity of the topic (e.g., woman’s abuse), which required extensive consultation and negotiation prior to roll-out.
Adoption	Adoption was positively impacted by: • Having staff and consumer representation throughout the program. Consumer representation was noted as a strong facilitator in this aspect. • Monitoring and benchmarking progress of the program KPIs. • Having Champions training targets, with a strategy to meet the target.	Adoption was negatively impacted by: • The BPSO program being nursing-driven and therefore not perceived as having multidisciplinary application, which made it difficult to engage with other clinicians. • Poor documentation and/or tracking of progress and KPIs including Champions training targets. • Time required in planning a BPG meant that the roll-out time (e.g., time to train sufficient number of Champions) was limited.
Appropriateness	Appropriateness was positively impacted by: • Choosing the correct BPGs to align with the strategic directions of the organization • Having a communication plan to support the BPSO program and including it in induction and education allowed staff to understand how it benefited the organization, their roles and their patients/clients	Appropriateness was negatively impacted by: • Lack of promotion and visibility of trained champions, the work they do, how it stemmed from the BPSO program and of the BPSO program as a whole, which affected people’s perceptions of whether it was useful, important or necessary endeavor to support.
Cost/resources	Cost/resources was positively impacted by: • Externally sourced funding attached a budget line to the program and protected the core requirements to deliver on the program. • Utilizing internal resourcing, partnerships, and activities to optimize efficient delivery of the program within the local context. • Having initial resources to fund the program, which was perceived as essential for its viability and legitimacy.	Cost/resources was negatively impacted by: • Lack of inflexibility to internal annual accounting requirements (end of financial year acquittal), which placed unnecessary levels of budget planning and negotiation to justify use of resources across the three years, so as not to lose the committed funds.
Feasibility	Feasibility was positively impacted by: • Having the organization in a position with clear strategies as to where it was heading and knowing how the guidelines would fit with the strategies. • Having organizational executive leadership and support, a designated BPSO site lead, and the Australian host (ANMF (SA Branch)) support (all perceived as critical elements).	Feasibility was negatively impacted by: • Releasing staff for champions training. This may have been as a result of timing of training sessions or reported as a result of insufficient backfill or staff levels on the day. This had implications for achieving 15% coverage across all areas. • Mixed levels of support and uptake of the program including champions training by executive, clinical staff, and consumer input. • High levels of staff and management turnover (churn), and challenges with overall staffing numbers.
Fidelity	Fidelity was positively impacted by: • The relatively prescribed approach of the BPSO program and its KPIs. Examples of fidelity measures include: establishment of the BPSO steering committee; identification of non-compliance with evidence based best practice (gap analysis); utilization of BPGs to inform practice, training and recruitment of Champions; and BPG implementation reporting and evaluation (action plan).	Fidelity was negatively impacted by: • Having ‘noise’ of other training programs or other initiatives that were very similar to the objectives of the selected BPGs, which then made it difficult for site leads to either work with those other initiatives to align the training with the BPG and meet their KPIs • Poor maintenance and lack of commitment of expected tasks associated with the BPSO program impacted on the quality of much of the planning work required for successful execution.
Penetration	Penetration was positively impacted by: • Having an executive sponsor for the program. This was noted as critical because executive support was critical to reminding people of the program and keeps it on the organization’s agenda at a broad level. • Having a BPSO program KPI of engaging a critical mass of 15% of nursing and midwifery staff in clinical settings as champions where guidelines are being implemented. • Establishing a champions and super champions network with meetings occurring monthly and quarterly, respectively.	Penetration was negatively impacted by: • Turnover and movement of staff within the network, which meant that trained Champions were lost and therefore impacted momentum and consistency, as well as BPSO KPIs. • Maintaining an active network of champions or super champions.
Sustainability	Sustainability was positively impacted by: • Establishing the right governance and BPSO steering committee membership, which was perceived to also ensure long-term sustainability beyond the pre-designate period. • Situating the BPSO program within a clinical unit that is agile, guided by transformational leadership, has capacity to provide long-term commitment to the program and can serve as program ambassadors. • Having a sustainability plan inclusive of strategies related to maintenance, program growth, risk management, embedding BPSO related work into everyday practice, and aligning the program’s KPIs with those of the organization.	Sustainability was negatively impacted by: • Turnover in executive and senior staff impacted on continuity of commitment and championing of the program at executive level discussion. • Not having a sustainability plan with a component focused on embedding and aligning the work with everyday practice. • Not aligning BPSO program KPIs to organizational KPIs related to safety and quality, education or practice improvement.

**Table 4:** Description of downstream impact on service outcome of the BPSO program.

Service Outcome Domain	Notes on Impact on Service Outcome
Effective	• Effectiveness of the program was assessed against the BPGs and the affiliated studies and audits. Their use in practice was demonstrated in practice changes such as the Person Centered KPI Project (various wards), woman screening, consumer feedback survey, and care transitions bedside handover consumer survey. • BPSO nursing indicators were well-defined for readily measurable clinical events such as falls or restraint usage, however for some BPGs there were no measurable indicators to report against; consequently, these had to be defined and worked out by the site.
Efficient	• Facilitator of efficiency was inferred from the strategic selection of BPGs that aligned with relevant works and committees such as the NSQHS standards and Consumer and Community Engagement Strategy. • Progress and performance reporting and the data systems used were not lean and involved various data collection methods, duplicative data entry, and support from multiple stakeholders to provide the necessary data. • Efficiency through cost saving associated with initiatives associated with the BPSO program were reported. For example, the chaperone program has reduced reliance on agency and security guard use, both of which are a more expensive workforce to maintain.
Equitable	• There was a deliberate approach reported to ensure equity across the program and the three BPGs. Examples of activities included: stakeholder engagement across culturally and linguistically diverse (CALD) people, migrant health service and aboriginal health service, and health literacy work capacity building through workshops/online courses specifically designed for Aboriginal persons, i.e., Understanding and Managing Risk: Domestic and Aboriginal Family Violence Training 1st July 2016, Aboriginal Cultural Learning online course, Kaurna Cultural Tour, and Champions workshop with the Aboriginal Educator Development officer. Demonstration of stakeholder assessments for BPGs, i.e., CALD People; migrant health service and aboriginal health service. • Some initiatives implemented as part of the BPSO program may be inherently addressing equity of care. For example, the Maastricht interview training program reported as part of the PFCC BPG may be building staff capacity to engage with, and subsequently provide better care to, people who have auditory hallucinations.
Patient-centered	• Patient-centeredness was a core component of the three BPGs selected. The Consumer and Community engagement team were actively involved in all aspects of the PFCC BPG; from governance to workshop participation, to evaluation activities. Key projects included the Person Centered KPI Project and initiatives such as Shared Decision Making, the Health Literacy Fact sheet series, and Consumer Input and Feedback. Engagement and partnership across the network occurred at all levels with the Consumer and Community Engagement Division. • Consumer co-delivery of workshops, preparing and reviewing literature, involvement in designing, and evaluating data and engaging in multi-disciplinary conversation around how to improve the program was noted as a strength of the program. • Training and education around the Champion training included reflective exercises for staff that has been meaningful as documented in workshop evaluations. There were shifts, because of the BPSO program, around the way staff work with consumers especially around choice, option talk, and decision making. The BPSO program encouraged choice- and decision- based conversations with patients/clients, which was a deliverable for the PFCC BPG within the organization.
Safe	• BPG activities have been designed to align to safety and quality initiatives that meet the NSQHS standards. • A key element of the BPG Care Transitions and Woman Abuse has been safety. Integrating safety into these BPGs has included working with and actioning various activities through relevant committees. Training and resources generated have reflected the input of these committees regarding safety and quality. • One of the eight KPIs in the Person Centered KPI project was: Patient’s sense of safety whilst under the care of the nurse/midwife and was assessed by asking patients and observed practice. This outcome was approximately 90% (always) across five wards and 75% (always), and 18% (most of the time), for the other ward. Over the 2–3-year auditing cycle there was demonstrable improvements across several of the KPIs. • Training and funded projects under the suicide prevention BPG and alternative use of restraints BPG were centered around client safety, as well as person-centered care and human rights.
Timely	• An example of timeliness related to the care transitions BPG at Site 1. Identified gaps in practice and changes addressed as part of this BPG include; timeframe for handover, enabling timely discharge, and the development of the General Practice Liaison Unit (GPLU), which was developed to address communication from hospital to doctors in the community.
